# CKD Progression and Economic Burden in Individuals With CKD Associated With Type 2 Diabetes

**DOI:** 10.1016/j.xkme.2022.100532

**Published:** 2022-08-11

**Authors:** C. Daniel Mullins, Kevin M. Pantalone, Keith A. Betts, Jinlin Song, Aozhou Wu, Yan Chen, Sheldon X. Kong, Rakesh Singh

**Affiliations:** 1 University of Maryland Baltimore, Baltimore, MD; 2 Department of Endocrinology and Metabolism, Cleveland Clinic, Cleveland, OH; 3 Analysis Group, Inc, Los Angeles, CA; 4 Bayer U.S. LLC, Whippany, NJ

**Keywords:** Type 2 diabetes, chronic kidney disease, CKD progression, healthcare resource utilization, healthcare costs

## Abstract

**Rationale & Objective:**

To evaluate progression patterns and associated economic outcomes, using estimated glomerular filtration rate (eGFR) and urine albumin-creatinine ratio (UACR) based on the Kidney Disease: Improving Global Outcomes (KDIGO) risk categories, among patients with type 2 diabetes (T2D) and chronic kidney disease (CKD).

**Study Design:**

Patients with T2D and moderate- or high-risk CKD were selected from the Optum electronic health records database (January 2007-December 2019). Progression patterns and post-progression economic outcomes were assessed.

**Setting & Participants:**

Adults with T2D and CKD in clinical settings.

**Predictor:**

Baseline KDIGO risk categories.

**Outcomes:**

Progression to a more severe KDIGO risk category; healthcare resource utilization and medical costs.

**Analytical Approach:**

Progression probability was estimated using cumulative incidence. Healthcare resource utilization and costs were compared across progression groups.

**Results:**

Of 269,187 patients (mean age 65.6 years) with T2D and CKD of moderate or high baseline risk, 18.9% progressed to the very high-risk category within 5 years. Among moderate-risk patients, 17.8% of CKD stage G1-A2, 44.0% of stage G2-A2, and 61.3% of stage G3a-A1 patients progressed to a higher KDIGO risk category. Among high-risk patients, 63.9% of stage G3b-A1/G3a-A2 and 56.0% of stage G2-A3 patients progressed to very high risk. Within the same eGFR stage, a higher UACR stage was associated with 4- to 7-times higher risk of progressing to very high risk and faster eGFR decline. Nonprogressors had lower annual medical costs ($16,924) than patients who progressed from moderate risk to high risk ($22,117, *P* < 0.05), from high risk to very high risk ($32,204, *P* < 0.05), and from moderate risk to very high risk ($35,092, *P* < 0.05).

**Limitations:**

Infrequent lab testing might have caused lags in identifying progression; medical costs were calculated using unit costs.

**Conclusions:**

Patients with T2D and CKD of moderate or high risk per KDIGO risk categories had high probabilities of progression, incurring a substantial economic burden. The results highlight the value of UACR in CKD management.

## Visual Abstract

**Figure:**
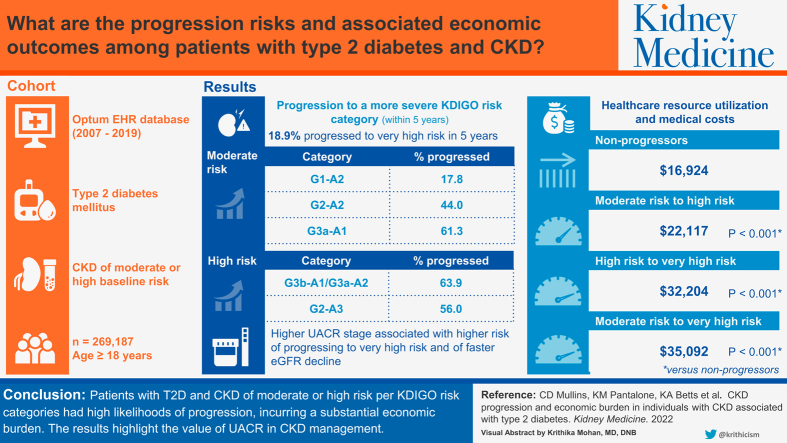

**Plain-Language Summary**

The Kidney Disease: Improving Global Outcomes (KDIGO) clinical guidelines characterize chronic kidney disease (CKD) prognostic risk categories using both glomerular filtration rate and urine albumin-creatinine ratio (UACR). Using a US electronic medical records database of patients with type 2 diabetes (T2D), this study assessed the risk of CKD progression in KDIGO risk categories and quantified the economic burden by progression group. We found that patients with T2D and CKD at moderate or high risk per KDIGO risk categories, especially those with impaired UACR, had high probabilities of CKD progression. In addition, patients who progressed incurred a substantial economic burden. Taken together, these results underscore the high burden associated with CKD progression and highlight the value of UACR in CKD management.

Diabetes is the leading cause of chronic kidney disease (CKD), which is characterized by low estimated glomerular filtration rate (eGFR) and high albuminuria.^1^ Approximately 1 in 3 US adults with type 2 diabetes (T2D) have CKD (6-10 million people).^2^, ^3^, ^4^ CKD with diabetes is associated with substantial morbidity and higher risk of severe complications and death.^2^ CKD management poses a substantial economic burden to patients and health systems, and healthcare resource utilization (HRU) and medical costs increase exponentially as the severity of CKD increases.^5^, ^6^, ^7^, ^8^ Thus, a major treatment goal for this patient population is the prevention of disease progression.^9^

The most widely used system of CKD staging and progression is based on lab tests of glomerular function, particularly eGFR.^10^, ^11^, ^12^, ^13^, ^14^, ^15^ However, this method does not incorporate another important domain of kidney health, glomerular damage, which is usually represented by albuminuria and indicated by an elevated urine albumin-to-creatinine ratio (UACR).^16^^,^^17^ Elevated UACR is an early marker of abnormal kidney function among patients with T2D, even when eGFR remains normal.^18^, ^19^, ^20^, ^21^, ^22^, ^23^ It is associated with higher risks of morbidity, mortality, and kidney failure and is an independent predictor of poor cardiovascular outcomes.^24^, ^25^, ^26^, ^27^, ^28^ Thus, UACR measurement is used to inform fundamental clinical decisions, such as whether to refer to a nephrologist, perform a kidney biopsy, or initiate therapy.^29^ The Kidney Disease: Improving Global Outcomes (KDIGO) clinical guidelines characterize CKD prognostic risk categories using both UACR stage (A1-A3) and eGFR stage (G1-G5).^16^ However, the disease progression patterns defined by KDIGO risk categories and the associated economic outcomes have not been quantified among patients with CKD and T2D.

An improved, more holistic understanding of the clinical course of CKD progression, measured using both eGFR and UACR, can inform clinical practice for patients with T2D by identifying patterns associated with fast disease progression. Thus, this study described the CKD progression patterns based on KDIGO risk categories and the associated HRU and medical costs among patients with CKD and T2D in the US.

## Methods

### Data Source

This retrospective cohort study included adults (aged greater than or equal to 18 years) with CKD and T2D in the Optum electronic healthcare records database from January 2007 to December 2019. The Optum electronic healthcare records database represents more than 150,000 medical providers from over 2,000 hospitals and 7,000 clinics and is comprised of medical records containing information on demographic characteristics, medical history and diagnoses, procedures, medications, and laboratory tests. As the data were deidentified, no institutional board review and informed consent were required.

### Study Population and Design

Adults with T2D and CKD of moderate (G3a-A1 and G1/2-A2) or high risk (G3b-A1, G3a-A2, G1/2-A3) based on the KDIGO heat map were identified in the database.^30^ Patients with T2D were identified based on a modified version of the Electronic Medical Records and Genomics algorithm, using *International Classification of Diseases, Ninth* and *Tenth Revisions* (ICD) diagnosis codes, T2D medications, and abnormal glucose (≥7.0 mmol/L [fasting glucose] or ≥11.1 mmol/L [random glucose]) or hemoglobin A1c (HbA1c) (≥6.5%) (Table S1).^31^ Patients with CKD were identified as having 2 reduced eGFR measurements (<60 mL/min/1.73 m^2^) 90-365 days apart and/or one increased UACR measurement (≥30 mg/g)^2^ after T2D diagnosis. eGFR was calculated using serum creatinine lab results per the 2009 CKD Epidemiology Collaboration creatinine equation.^32^ UACR measures included UACR test results or a calculation based on urine albumin and urine creatinine tests (measured within 24 hours) if an UACR test was unavailable. The KDIGO risk category was determined based on lab results only, using 2 eGFR measures 90-365 days apart, indicating the same eGFR stage, and one UACR measure obtained ≤1 year of the established eGFR stage. eGFR levels were classified into 6 G stages: normal or high (G1, ≥90 mL/min/1.73 m^2^); mildly decreased (G2, 60-89 mL/min/1.73 m^2^); mild to moderately decreased (G3a, 45-59 mL/min/1.73 m^2^); moderately to severely decreased (G3b, 30-44 mL/min/1.73 m^2^); severely decreased (G4, 15-29 mL/min/1.73 m^2^); and kidney failure (G5, <15 mL/min/1.73 m^2^). UACR levels were classified into 3 A stages: normal to mildly increased (A1, <30 mg/g); moderately increased (A2, 30-300 mg/g); and severely increased (A3, >300 mg/g). Because of infrequent UACR measurements in the study population, only one UACR measure was used to establish KDIGO risk category.

For the CKD progression analysis, the index date was defined as the earliest record indicating CKD of moderate or high risk after T2D diagnosis. The follow-up period spanned from the index date to the earliest of the end of continuous eligibility or the end of data availability. In the analysis of HRU and healthcare costs, the index date was the date of the earliest record indicating CKD progression for those who progressed, or the earlier of 2 years before the end of follow-up and the date of patients’ first identified KDIGO risk categories for those who did not progress. The study period was 2 years post index date, and therefore, the patient follow-up time in the HRU and costs analysis were capped at 2 years. The baseline period encompassed the 6 months before the index date.

Patients meeting the aforementioned inclusion criteria were included if they (1) had continuous enrollment during the 6-month baseline period; (2) did not have kidney failure, dialysis, kidney transplantation, or other types of diabetes at study baseline; (3) had a sufficient number of eGFR and UACR tests with least one KDIGO risk category identified during the follow-up period; and (4) had CKD of moderate or high risk per the KDIGO heatmap at index.

### CKD Progression

CKD progression was defined as a change from moderate risk to high/very high risk or from high risk to very high risk, per KDIGO heatmap, based on the most severe risk category a patient ever experienced within 5 years post index. The very high-risk group included G4/5-A1/3, G3b-A2/3, and G3A-A3. Only one progression event, if any, was counted per patient. Patients without progression who moved from a higher to a lower risk category were included in the no progression group. To visualize the 5-year eGFR trajectory, eGFR values were calculated as the average of measurements ±60 days each year at the end of years 1-5.

### Economic Outcomes

All-cause HRU assessed during the study period included inpatient admissions, emergency room (ER) visits, and outpatient visits. All-cause medical costs (inpatient, ER, and outpatient costs; in 2020 US dollars) were estimated using a unit-costing approach by multiplying the frequencies of each HRU component with the corresponding unit costs generated from the Optum Clinformatics claims data. CKD-related medical costs were defined as costs associated with diagnosis for CKD or related diseases and complications (acute kidney injury, anemia, hyperkalemia, metabolic acidosis, obesity, hypertension, hyperlipidemia, cardiovascular disease, and cerebrovascular diseases) and were calculated using the same unit-costing approach.

### Statistical Analyses

#### Baseline Characteristics

Patient characteristics were summarized over the baseline period by index risk category for the CKD progression analysis and by CKD progression pattern for the HRU and costs analysis. For the CKD progression analysis, the baseline characteristics of high-risk patients were compared with those of moderate-risk patients. For the HRU and costs analysis, the baseline characteristics of progressors were compared with those of non-progressors.

#### CKD Progression

For each index KDIGO risk category, the 5-year probability of moving to a more severe risk category (ie, from moderate to high/very high risk and from high to very high risk) was estimated. Different ending risk categories were treated as different outcomes. Because movements to different ending risk categories were treated as mutually exclusive events, competing risks existed between these events. Thus, the probability of moving to a risk category was estimated using cumulative incidence (derived based on cause-specific hazards for the corresponding outcome and event-free survival for all outcomes) to address the competing risk of moving to the other risk categories.^33^ The projected distribution of KDIGO risk categories by year 5 post index was estimated. For each risk cell, the projected proportion of patients in the risk cell equaled the sum of the estimated proportion remaining in the risk cell and the estimated proportion that progressed to this risk cell from all others. These 2 quantities were derived from the baseline KDIGO risk category distribution and the estimated 5-year progression probability for each risk cell. Changes in kidney function over time were assessed using the trajectories of eGFR, which were depicted by line charts using mean eGFR values at the index date and at years 1-5 post index by index KDIGO risk category.

#### Economic Outcomes

HRU and costs were summarized by progression patterns (ie, moving from moderate to high/very high risk or from high to very high risk). Frequencies of medical services and days of inpatient stay were annualized (per patient per year) to account for patients’ varying follow-up lengths and summarized using means and standard deviation. The number of patients with greater than or equal to 1 inpatient admission was summarized using frequency and percentages. Statistical comparisons were conducted for each progression risk category with the no progression group using Wilcoxon rank-sum tests for continuous variables and χ^2^ tests for categorical variables.

SAS software (v9.4; SAS Institute) and R software (v3.6.3; the R Foundation) were used for statistical analyses. All statistical tests were two-sided, and *P* < 0.05 was considered significant.

## Results

### Patient Characteristics

**Figure 1:**
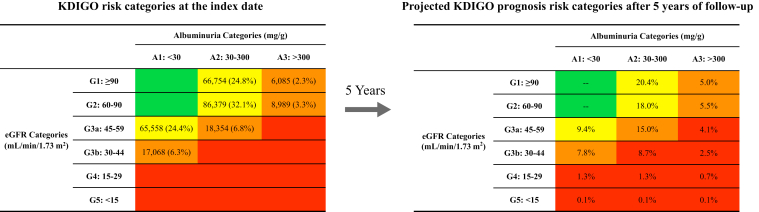
Distribution of KDIGO prognosis risk categories at the index date and 5 years post index date. Abbreviation: eGFR, estimated glomerular filtration rate; KDIGO, Kidney Disease: Improving Global Outcomes.

A total of 269,187 patients with CKD and T2D met the study criteria and were included in the analyses (Fig S1). At the index date, 81.2% (n = 218,691) of patients were moderate risk and 18.8% (n = 50,496) were high risk per KDIGO criteria (Table 1). The most populated risk category was G2-A2 (32.1%) followed by G1-A2 (24.8%) and G3a-A1 (24.4%) (Fig 1).

**Table 1:** Patient Baseline and Clinical Characteristics

	All Patients N = 269,187	Index KDIGO Risk Category		*P* (High vs Moderate Risk)
		Moderate N = 218,691	High N = 50,496	
**Demographics, n (%)**				
Age (y), mean ± SD	65.6 ± 12.2	64.7 ± 12.1	69.5 ± 11.8	< 0.001^a^
Male	130,171 (48.4%)	106,306 (48.6%)	23,865 (47.3%)	< 0.001^a^
US region				< 0.001^a^
Midwest	153,948 (57.2%)	125,956 (57.6%)	27,992 (55.4%)	
South	61,403 (22.8%)	49,127 (22.5%)	12,276 (24.3%)	
Northeast	31,011 (11.5%)	25,323 (11.6%)	5,688 (11.3%)	
West	17,179 (6.4%)	13,769 (6.3%)	3,410 (6.8%)	
Other/unknown	5,646 (2.1%)	4,516 (2.1%)	1,130 (2.2%)	
Race				< 0.001^a^
White	221,580 (82.3%)	179,687 (82.2%)	41,893 (83.0%)	
African American	27,583 (10.2%)	22,422 (10.3%)	5,161 (10.2%)	
Asian	5,642 (2.1%)	4,780 (2.2%)	862 (1.7%)	
Other/unknown	14,382 (5.3%)	11,802 (5.4%)	2,580 (5.1%)	
**Lab tests, mean ± SD**				
Index eGFR (mL/min/1.73 m^2^)	73.7 ± 22.6	77.4 ± 21.1	57.9 ± 22.2	< 0.001^a^
Index UACR (mg/g)^b^	38.9 (20.0, 73.1)	37.7 (20.4, 63.0)	56.0 (18.4, 332.4)	< 0.001^a^
HbA1c (%)^c^	7.3 ± 1.5	7.3 ± 1.5	7.3 ± 1.5	0.06
**Comorbidities, n (%)**				
Hypertension	181,780 (67.5%)	145,182 (66.4%)	36,598 (72.5%)	< 0.001^a^
Hyperlipidemia	168,375 (62.5%)	136,787 (62.5%)	31,588 (62.6%)	0.98
Obesity	47,159 (17.5%)	38,971 (17.8%)	8,188 (16.2%)	< 0.001^a^
Ischemic heart disease	44,198 (16.4%)	33,227 (15.2%)	10,971 (21.7%)	< 0.001^a^
Chronic pulmonary disease	39,250 (14.6%)	30,928 (14.1%)	8,322 (16.5%)	< 0.001^a^

Abbreviations: eGFR, estimated glomerular filtration rate; HbA1c, hemoglobin A1c; KDIGO, Kidney Disease: Improving Global Outcomes; SD, standard deviation; UACR, urine albumin-to-creatinine ratio; US, United States.

a *P* < 0.05.

b Median (interquartile range) was presented for UACR.

c HbA1c was reported among 269,187 patients without missing value.

Baseline characteristics in the overall cohort and by risk category are presented in Table 1. The study population had a mean age of 65.6 (standard deviation 12.2), with 51.6% female. Compared with patients with CKD of moderate risk, high-risk patients were older (69.5 [standard deviation 11.8] vs 64.7 [12.1] years), had lower index eGFR (57.9 [22.2] vs 77.4 [21.1] mL/min/1.73 m^2^), higher UACR (median 56.0 [interquartile range, 18.4-332.4] vs 37.7 [20.4-63.0] mg/g), and higher prevalence of hypertension (72.5% vs 66.4%), ischemic heart disease (21.7% vs 15.2%), and chronic pulmonary disease (16.5% vs 14.1%), as well as slightly lower prevalence of obesity (16.2% vs 17.8%; all *P* < 0.001).

### Five-year CKD Progression

**Figure 2:**
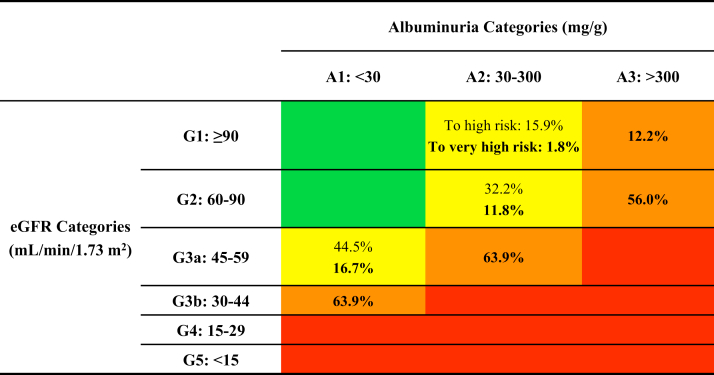
Five-year probability of progression to the high or very high-risk categories by index KDIGO risk category. Abbreviations: eGFR, estimated glomerular filtration rate; KDIGO, Kidney Disease: Improving Global Outcomes. Note: Bolded numbers represent the probability of progression to the ‘very high risk’ category.

Patients with CKD of moderate or high risk defined by baseline KDIGO risk categories had high probabilities of moving to higher risk categories within 5 years (Figs 1 and 2). The majority of high-risk patients were projected to move to the very high-risk category (G3b-A1/G3a-A2: 63.9%, G2-A3: 56.0%) except for G1-A3 (12.2%). The probability of moving to a higher risk category was also high for patients with moderate risk at baseline (ie, G1-A2: 17.8%; G2-A2: 44.0%; G3a-A1: 61.3%). For patients in the same eGFR stage, a higher UACR stage was associated with a 4- to 7-times higher risk of moving to the very high-risk category (eg, G2-A2 [11.8%] vs G2-A3 [56.0%]; G3a-A1 [16.7%] vs G3a-A2 [63.9%]). By the end of year 5, 18.9% of patients in any index risk category were projected to progress to the very high-risk category (Fig 1).

### eGFR Trajectory

**Figure 3:**
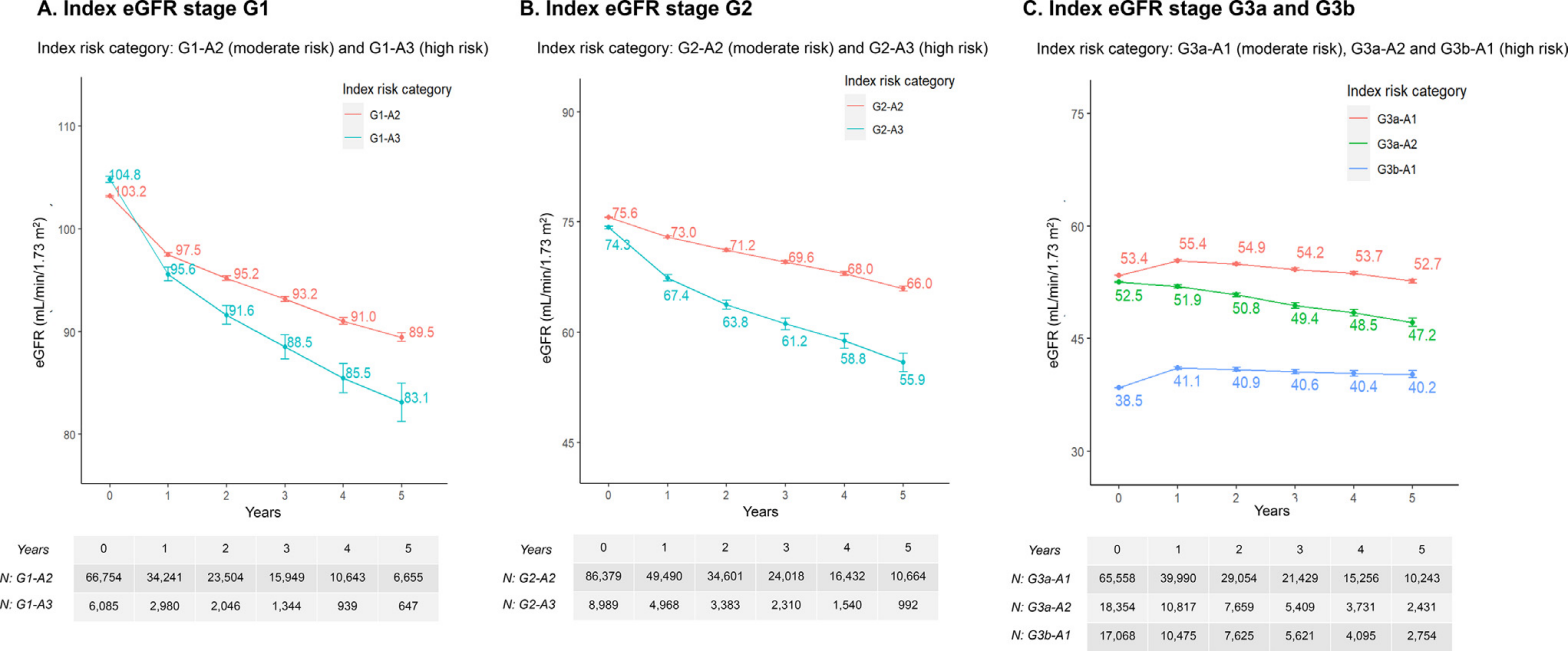
Mean eGFR trajectories in patients with chronic kidney disease of moderate or high risk on the index date. Abbreviation: eGFR: estimated glomerular filtration rate.

eGFR levels decreased over time among all patients. For patients with the same index eGFR stage, those with higher UACR values generally experienced a faster decline in eGFR over 5 years (Figure 3A-C). For example, G1-A3 patients experienced a faster 5-year eGFR decline (difference in eGFR: 21.7 mL/min/1.73 m^2^) than G1-A2 patients (13.7 mL/min/1.73 m^2^), and G2-A3 patients experienced a faster 5-year eGFR decline (18.4 mL/min/1.73 m^2^) than G2-A2 patients (9.6 mL/min/1.73 m^2^).

### All-cause HRU and Medical Costs

A total of 269,085 patients with at least 1 day of follow-up were included in the analyses of HRU and medical costs (Table S2). Among these patients, 209,756 experienced no progression; 41,986 moved from the moderate to high-risk category; 3,102 moved from the moderate to very high-risk category; and 14,241 moved from the high to very high-risk category.

Patients who progressed to a higher risk category had more all-cause inpatient, ER, and outpatient visits and longer inpatient stays per year during the follow-up period (mean 1.36 to 1.62 years across groups) than those who did not progress (Table 2). For example, patients who progressed from the high to very high-risk category had over twice was many annual inpatient admissions (0.71 vs 0.32) and inpatient days (5.54 vs 2.34) as well as significantly more ER visits (0.62 vs 0.45), than patients who did not progress (all *P* < 0.001).

**Table 2:** Annual All-cause HRU by CKD Progression Category

	No Progression N = 209,756	CKD Progression Pattern					
		Moderate to High N = 41,986		Moderate to Very High N = 3,102		High to Very High N = 14,241	
			*P*^a^		*P*^a^		*P*^a^
**Years of follow-up post-progression, mean ± SD**	1.62 ± 0.55	1.48 ± 0.66	< 0.001^b^	1.36 ± 0.70	< 0.001^b^	1.45 ± 0.67	< 0.001^b^
**Inpatient admissions**							
Patients with ≥1 admission, n (%)	46,423 (22.13%)	11,524 (27.45%)	< 0.001^b^	1,143 (36.85%)	< 0.001^b^	4,944 (34.72%)	< 0.001^b^
Number of admissions (PPPY), mean ± SD	0.32 ± 1.10	0.43 ± 1.35	< 0.001^b^	0.77 ± 2.46	< 0.001^b^	0.71 ± 2.27	< 0.001^b^
Days of stay (PPPY), mean ± SD	2.34 ± 11.00	3.21 ± 14.79	< 0.001^b^	6.00 ± 20.46	< 0.001^b^	5.54 ± 19.24	< 0.001^b^
**ER visits**							
Patients with ≥1 visit, n (%)	63,765 (30.40%)	12,995 (30.95%)	0.03^b^	1,098 (35.40%)	< 0.001^b^	4,798 (33.69%)	< 0.001^b^
Number visits (PPPY), mean ± SD	0.45 ± 1.32	0.50 ± 1.94	0.002^b^	0.68 ± 1.73	< 0.001^b^	0.62 ± 3.12	< 0.001^b^
**Outpatient visits**							
Patients with ≥1 visit, n (%)	208,375 (99.34%)	41,560 (98.99%)	< 0.001^b^	3,059 (98.61%)	< 0.001^b^	14,014 (98.41%)	< 0.001^b^
Number of visits (PPPY), mean ± SD	18.57 ± 17.30	22.91 ± 19.94	< 0.001^b^	25.88 ± 23.55	< 0.001^b^	23.43 ± 19.88	< 0.001^b^

Abbreviations: CKD, chronic kidney disease; ER, emergency room; HRU, healthcare resource utilization; PPPY, per-person-per-year; SD, standard deviation.

a The *P*-value comparing each progression pattern with the no progression group

b *P* < 0.05.

**Figure 4:**
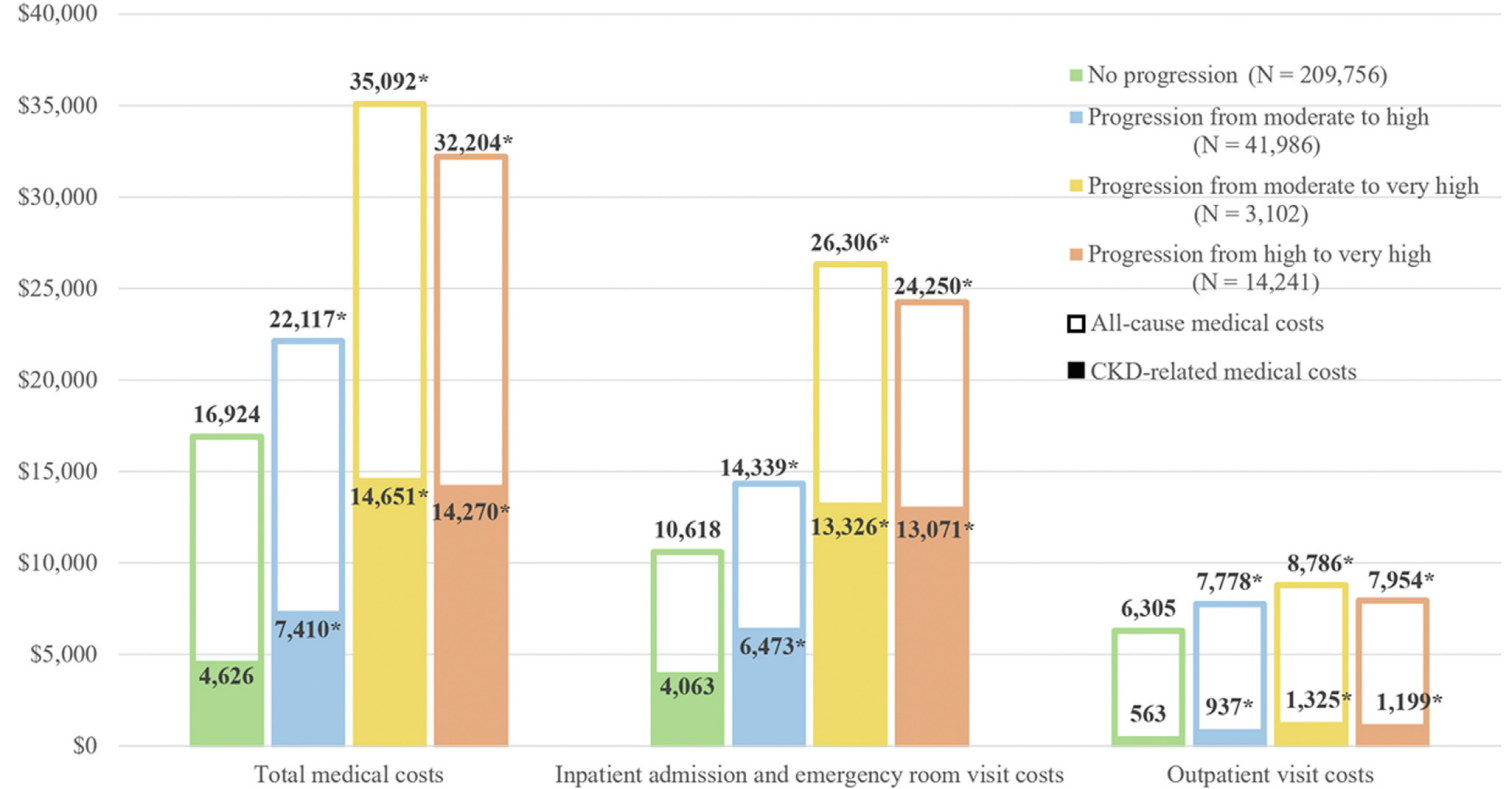
Average annual medical costs per patient, by progression category. Costs in 2020 United States dollars. ∗*P* < 0.001 compared with the no progression category. Abbreviation: CKD, chronic kidney disease.

Similar trends were observed for medical costs. Compared with the average annual all-cause medical costs of patients who did not progress ($16,924), significantly higher post-progression costs were incurred among those who moved from moderate to high risk ($22,117), from moderate to very high risk ($35,092), and from high to very high risk ($32,204; all *P* < 0.001) (Fig 4). Inpatient admission and ER visit costs were the major cost drivers across progression categories, constituting 62.7%-75.3% of total costs across different progression groups. CKD-related medical costs constituted 27.3% ($4,626) of the total medical costs for the no progression group and increased to 33.5% ($7,410) for patients progressing from moderate to high risk, 41.8% ($14,651) for patients progressing from moderate to very high risk, and 44.3% ($14,270) for patients progressing from high to very high risk (all *P* < 0.001 compared with the no progression group) (Fig 4).

## Discussion

The high prevalence and massive clinical and economic burdens associated with CKD and T2D in the United States underscore the importance of CKD management to avoid progression.^34^ The KDIGO guidelines recommended CKD prognosis risk categorization using both eGFR and UACR on a 2-dimensional heat map and annual monitoring of UACR.^16^^,^^17^^,^^23^ Despite these recommendations, the majority of clinical and real-world studies define CKD progression using eGFR alone. This large, real-world cohort of over 260,000 patients with both CKD and T2D in the United States assessed the patterns of progression (defined as advancing in the KDIGO risk categories) and observed a high probability of moving to higher risk categories within 5 years. Specifically, 19% of patients overall progressed to the very high-risk category within 5 years, with a high rate of progression observed in high-risk patients at baseline (eg, 63.9% of G3a/3b-A2 patients and 56.0% of G2-A3 patients moved to the very high-risk category).

A study by Levey et al^30^ demonstrated that patients with more severe KDIGO risk categories have higher risk of kidney failure, acute kidney injury, cardiovascular mortality, and all-cause mortality. Specifically, patients in the very high-risk category in that study experienced a 2- to 3-fold higher risk of all-cause mortality and 10- to 100-fold higher risk of developing kidney failure compared with patients of moderate risk.^30^ Therefore, the high progression rates observed in this study among patients with CKD and T2D translate to a high clinical burden in terms of morbidity and mortality.

Our study also indicated that UACR can further differentiate the risk of progression among patients within the same eGFR stage, which is a valuable insight given the consequences of progression. For patients within the same eGFR stage, having a higher UACR stage was associated with a 4- to 7-times higher risk of progressing to the very high-risk KDIGO category, and patients with higher UACR experienced faster decline in eGFR. These results are consistent with those of previous studies. For example, Meguro et al^35^ reported that patients with T2D and microalbuminuria or macroalbuminuria had over 10- and 130-fold increased risk of reaching eGFR <30 mL/min/173 m^2^, respectively, compared with patients with normoalbuminuria during a 3-year follow-up. Additionally, Leehey et al^36^ reported that having more severe proteinuria, a marker highly correlated with albuminuria, was associated with faster disease progression in diabetic patients with CKD. These findings confirm the value of UACR in evaluating CKD severity and emphasize the importance of UACR monitoring in adults with CKD and T2D, which would enable identification and targeting of patients at risk of rapid disease progression for earlier and perhaps more effective therapeutic interventions.

The consequences of CKD progression involve more intensive and frequent medical interventions, translating to higher HRU and costs. This study demonstrated that, compared with patients without CKD progression, those who progressed to more severe KDIGO risk categories incurred significantly more HRU and medical costs. The no progression group had, on average, $15,000-$18,000 lower medical costs compared with patients who progressed to the very high-risk group, and even patients who progressed to the high-risk group still had costs that were ∼$10,000 lower than those who progressed to the very high-risk group. Across risk categories, costs related to inpatient admissions and ER visits were the main drivers of the incremental costs, and CKD-related costs accounted for 42%-44% of the total costs in patients who progressed to the very high-risk category. These results are consistent with previous studies which used eGFR alone to define the severity of CKD. Golestaneh et al^6^ reported that mean annualized all-cause costs increased exponentially with advancing CKD stage. Similar results that inpatient costs were the key driver of the high medical costs were reported by Nichols et al.^37^

UACR is largely under-tested in real world practice, even among patients already diagnosed with CKD and T2D, despite clinical guideline recommendations that both eGFR and albuminuria should be measured at least annually in patients with CKD and more frequently in advanced CKD stages.^38^, ^39^, ^40^, ^41^, ^42^^,^^43^ The suboptimal adherence to clinical guidelines for CKD management highlight the need to improve patient and physician awareness regarding the importance of adequate UACR testing to avoid poor outcomes. Furthermore, the substantial clinical and economic burdens of patients with CKD and T2D, as well as the escalating incidence of these diseases in the United States, create the imperative to find novel and more effective therapies to slow progression and reduce the costs of CKD management. Sodium-glucose transport protein 2 inhibitors and the recently approved nonsteroidal mineralocorticoid receptor antagonist finerenone have demonstrated kidney- and heart-protective effects, which may reduce CKD-related hospitalizations.^44^^,^^45^

A notable strength of this study was the use of a large-scale electronic healthcare records database permitted the selection of a large cohort of adults with both CKD and T2D that was representative of all US geographic regions and adult age groups. Additionally, this study assessed CKD progression defined according to the KDIGO risk categories, which has not been comprehensively assessed in other studies. The progression outcomes and their associated economic impacts have unique value for informing decision making by healthcare providers, payers, and policymakers. CKD and KDIGO risk categories were defined using eGFR and UACR laboratory measurements, which are more accurate than only using diagnosis codes.

The results of this study should also be considered in the light of several limitations. First, as with all electronic healthcare records database analyses, claims for services obtained outside of the healthcare network were not captured; coding inaccuracy/errors may have led to misclassification of patients with T2D identified with ICD codes. Second, measures of eGFR are highly variable, which might have resulted in misclassification of CKD risk categories. To reduce the possibility of misclassification, 2 measures of eGFR were used to define G stage. Third, as CKD progression was defined as advancing in KDIGO risk categories, the possible progression outcomes rely on the initial risk categories. For example, patients in G1-A3 can only progress to the very high-risk group by advancing in eGFR categories. Fourth, infrequent testing of kidney function, especially UACR, might have caused lags in disease progression identification. Because of infrequent UACR testing in the population, only one UACR measure was used to establish KDIGO risk category, which might have led to misclassification. Fifth, medical costs were calculated using unit costs generated from a separate claims database; therefore, the approach may not capture the actual costs incurred. In addition, the CKD-related cost estimation is prone to potential measurement error as it relies on the completeness of included CKD-related complications and comorbid conditions as well as the sensitivity and specificity of diagnosis and procedure codes used to identify them. In addition, this study did not evaluate patients with low-risk CKD per the KDIGO heatmap (G1-A1 and G2-A1) at baseline because using eGFR measurement alone has low specificity for identifying stage 1 and 2 CKD because of the difficulty of differentiating moderately decreased eGFR caused by CKD versus normal aging in elderly patients, which can lead to misclassification.^46^^,^^47^ CKD progression patterns among patients in the low-risk KDIGO category warrants future research. Finally, this is a descriptive study and the results do not imply any causal relationship. Future hypothesis-testing studies controlling for confounding are warranted.

In conclusion, in this retrospective study, patients with T2D and CKD who were in moderate or high KDIGO risk categories at the index date had high probabilities of progression to a higher risk category within 5 years. Moreover, an impaired UACR was associated with faster progression. Patients who progressed to a higher risk category incurred significantly higher HRU and medical costs compared with those without progression. These results underscore the high clinical and economic burdens associated CKD progression defined using both eGFR and UACR and highlight the value of UACR in CKD management.
