# Supplementary material for: CKD Progression and Economic Burden in Individuals With CKD Associated With Type 2 Diabetes
Source: Kidney Med. 2022 Aug 11;4(11):100532. doi: 10.1016/j.xkme.2022.100532 (PMC9630787; doi:10.1016/j.xkme.2022.100532)
Supplement: Supplementary File (PDF) — Figure S1, Table S1-S2. [file mmc1.pdf]

Figure S1. Sample selection flowchart - patients with CKD of moderate or high risk associated with T2D

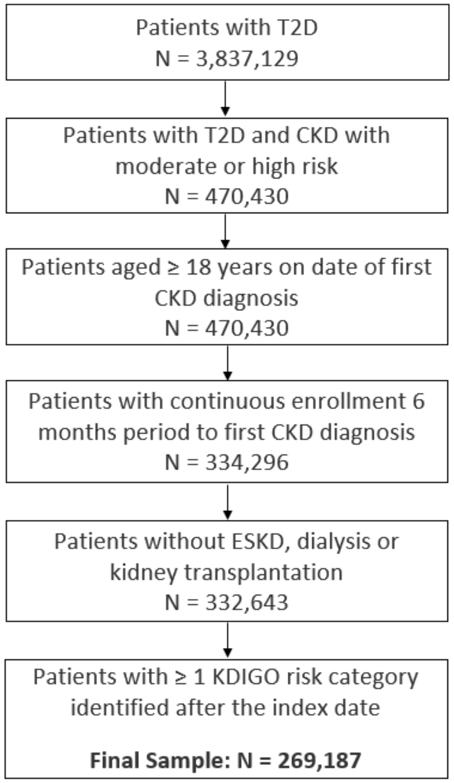

Abbreviations: CKD, chronic kidney disease; ESKD, end-stage kidney disease; KDIGO, Kidney Disease: Improving Global Outcomes; T2D, type 2 diabetes

Table S1. Diagnostic codes and test codes used in the study

	Diagnostic codes and treatment codes	
**Diagnosis**	**ICD-9-CM**	**ICD-10-CM**
Type 2 diabetes	250.x0, 250.x2 (excluding 250.10 and 250.12)	E11 (excluding E11.1)
Other types of diabetes (for exclusion)		
Type 1 diabetes	250.x1, 250.x3	E10
Diabetes due to underlying conditions	249	E08
Chemical induced diabetes		E09
Other specified diabetes		E13
CKD [a]		
Stage 1	585.1	N18.1
Stage 2	585.2	N18.2
Stage 3	585.3	N18.3
Stage 4	585.4	N18.4
Stage 5	585.5	N18.5
Unspecified	585.9	N18.9
Diabetic CKD (type 2 diabetes)	250.40, 250.42	E11.22
Hypertensive CKD	403, 403.00, 403.01, 403.10, 403.11, 403.90, 403.91, 404, 404.00, 404.01, 404.02, 404.03, 404.10, 404.11, 404.12, 404.13, 404.90, 404.91, 404.92, 404.93	I12, I12.0, I12.9, I13, I13.0, I13.1, I13.2, I13.10, I13.11
Anemia in CKD	285.21	D63.1
**Treatment**	**GPI**	
Metformin	27250050xxxxxx, 27992502xxxxxx, 2799500270xxxx, 2799600220xxxx, 2799600230xxxx, 2799700230xxxx, 2799700235xxxx, 2799700240xxxx, 2799800240xxxx, 2799800260xxxx, 2799900250xxxx	
Sulfonylurea	2720xxxxxxxxxx	
Thiazolidinedione	276070xxxxxxxx	
Meglitinide analogues	2728xxxxxxxxxx	
Alpha-glucosidase inhibitors	2750xxxxxxxxxx	
DPP4i	2755xxxxxxxxxx	
SGLT2i	2770xxxxxxxxxx	
GLP1ra	2717xxxxxxxxxx	
Insulin	2710xxxxxxxxxx, 97201xxxxxxxxx	
Other anti-diabetic treatments	27xxxxxxxxxxxx (excluding hypoglycemia medications 2730xxxxxxxxxx and the specific antidiabetic medications listed above)	

Abbreviations: CKD: chronic kidney disease; ICD-9/10-CM: International Classification of Diseases, 9th/10th edition, Clinical Modification; DPP4i, dipeptidyl peptidase-4 inhibitor; GLP1ra, glucagon like peptide 1 receptor agonists; GPI, generic product indicator; SGLT2i, sodium glucose co-transporter 2 inhibitors.

Note: [a] The diagnosis codes for CKD stages were only used to identify CKD-related costs and HRU.

Table S2. Patient characteristics at the index date for the HRU and cost analysis

		CKD Progression Pattern					
	**No progression** N = 209,756	**Moderate to High** N = 41,986		**Moderate to Very High** N = 3,102		**High to Very High** N = 14,241	
**Demographics, n (%)**	Estimate	Estimate	P-value [a]	Estimate	P-value [a]	Estimate	P-value [a]
Age, mean ± SD	65.7 ± 12.5	70.9 ± 10.5	< 0.001 *	72.0 ± 9.7	< 0.001 *	73.9 ± 9.6	< 0.001 *
Male	101,918 (48.6%)	20,261 (48.3%)	0.22	1,375 (44.3%)	< 0.001 *	6,554 (46.0%)	< 0.001 *
Race			< 0.001 *		< 0.001 *		< 0.001 *
African American	22,105 (10.5%)	3,792 (9.0%)		297 (9.6%)		1,375 (9.7%)	
Asian	4,675 (2.2%)	700 (1.7%)		45 (1.5%)		222 (1.6%)	
Caucasian	171,019 (81.5%)	35,777 (85.2%)		2,648 (85.4%)		12,054 (84.6%)	
Other/unknown	11,957 (5.7%)	1,717 (4.1%)		112 (3.6%)		590 (4.1%)	
**Lab tests, mean ± SD**							
eGFR (mL/min/1.73m^2)	76.9 ± 21.9	57.8 ± 18.8	< 0.001 *	40.3 ± 10.3	< 0.001 *	40.6 ± 9.7	< 0.001 *
UACR (mg/g) [†]	34.0 (14.0, 64.2)	50.0 (30.0, 232.2)	< 0.001 *	86.0 (39.7, 327.0)	< 0.001 *	86.0 (39.0, 366.3)	< 0.001 *

HbA1c (%)	7.2 ± 1.5	7.2 ± 1.4	0.002 *	7.2 ± 1.5	< 0.001 *	7.2 ± 1.3	< 0.001 *
**Comorbidities, n (%)**							
Hypertension	149,175 (71.1%)	32,426 (77.2%)	< 0.001 *	2,548 (82.1%)	< 0.001 *	11,414 (80.1%)	< 0.001 *
Hyperlipidemia	136,860 (65.2%)	28,923 (68.9%)	< 0.001 *	2,159 (69.6%)	< 0.001 *	9,722 (68.3%)	< 0.001 *
Obesity	42,673 (20.3%)	7,563 (18.0%)	< 0.001 *	668 (21.5%)	0.11	2,527 (17.7%)	< 0.001 *
Ischemic heart disease	35,480 (16.9%)	10,036 (23.9%)	< 0.001 *	948 (30.6%)	< 0.001 *	4,315 (30.3%)	< 0.001 *
Chronic pulmonary disease	33,240 (15.8%)	7,550 (18.0%)	< 0.001 *	724 (23.3%)	< 0.001 *	2,885 (20.3%)	< 0.001 *

*p<0.05.

† Median (interquartile range) was presented for UACR.

Abbreviations: eGFR, estimated glomerular filtration rate; HbA1c: hemoglobin A1c; KDIGO, Kidney Disease: Improving Global Outcomes; SD, standard deviation; UACR, urine albumin to creatinine ratio.

Note: [a] P-values comparing each progression pattern with the no progression group
